# Multi-Horizon Delay Asymmetry Prediction and Compensation for IEEE 1588 Precision Time Protocol on Copper and Fiber Links by Means of Ground-Truth Measurements

**DOI:** 10.3390/s26144335

**Published:** 2026-07-08

**Authors:** Junchao Wang, Shengping Xu, Chuwen Tang, Chen Yang, Jiayue Shen, Jianye Zhao

**Affiliations:** School of Electronics, Peking University, Beijing 100871, China; wangjc@stu.pku.edu.cn (J.W.); 2401213420@pku.edu.cn (S.X.); tangchuwen@stu.pku.edu.cn (C.T.); 2501112243@stu.pku.edu.cn (C.Y.); 2201213008@stu.pku.edu.cn (J.S.)

**Keywords:** IEEE 1588, Precision Time Protocol, delay asymmetry compensation, cesium atomic clock, clock synchronization, copper and fiber links, hardware timestamping, precision measurement, embedded timekeeping

## Abstract

IEEE 1588 Precision Time Protocol (PTP) synchronization accuracy is degraded by delay asymmetry in physical links. Accurate prediction and compensation of this asymmetry are essential for high-precision timekeeping. This paper presents a multi-horizon delay asymmetry prediction framework validated on a cesium-referenced testbed. The experimental platform integrates a cesium atomic clock (frequency stability $<5\times 10^{-12}$), a Syncedge C10 PTP analyzer, and an RK3568 embedded TimeReceiver, providing hardware-timestamped ground-truth measurements over 24 h periods on both copper and fiber links under real operating conditions. A unified multi-output encoder architecture is compared against per-horizon recurrent models and classical baselines to predict delay asymmetry at five future horizons (h∈{1,2,4,8,16}, corresponding to 0.125–2.0 s ahead). On copper links at an 8 s observation window, the unified model achieves a mean absolute error of 2.15 ns and an $R^{2}$ of 0.714 at the nearest horizon, statistically tied with the best per-horizon model (2.16 ns, $R^{2}=0.713$) while using 1.9–6.1× fewer total parameters (655 K vs. 1.2–4.0M). On fiber links, all models achieve comparable accuracy with an MAE of 2.16–2.18 ns. The method requires ground-truth labels from a calibrated 1PPS or cesium reference; both training and inference require the same physical link and environmental conditions. Generalization to other settings remains untested.

## 1. Introduction

### 1.1. Motivation

IEEE 1588 Precision Time Protocol (PTP) is widely deployed for sub-microsecond synchronization in smart grids, 5G fronthaul, and industrial automation [1,2,3]. PTP operates on the assumption of symmetric forward and backward path delays. In practice, physical-layer (PHY) asymmetry and oscillator drift introduce non-negligible delay differences, even in direct copper or fiber links. This asymmetry directly corrupts the TimeReceiver clock offset estimate and degrades synchronization accuracy. Accurately predicting this asymmetry—and compensating for it—is therefore a meaningful research objective, though achieving reliable compensation without ground-truth calibration remains an open challenge.

### 1.2. Limitations of Existing Methods

Existing compensation techniques have several drawbacks. Transparent clock (TC) switches require specialized hardware and are inapplicable to direct links [4]. Packet selection algorithms discard most measurements and target network queuing rather than static or slowly varying PHY asymmetry [5]. Heuristic methods such as fuzzy-PI controllers [6,7,8] and Kalman filter-based approaches [9] rely on careful manual tuning and simplified noise assumptions. All existing approaches predict only a single immediate future value. In practice, PTP systems benefit from knowing the predicted asymmetry at multiple future horizons for adaptive control strategies, robustness to missed PTP exchanges, and multi-rate servo loops. The multi-horizon prediction problem for PTP delay asymmetry has received little attention in the literature.

### 1.3. Transformer for Multi-Horizon Prediction

The conventional per-horizon approach trains a separate model for each prediction horizon, which incurs a linear increase in parameter count with the number of horizons. A more parameter-efficient alternative is the multi-output topology: a single shared encoder processes the input sequence, and independent horizon-specific decoder heads produce predictions from the shared representation. This design principle applies to any encoder architecture—including LSTM, GRU, and Transformer. In this paper, we adopt a Transformer encoder with a multi-headed output layer [10]. Multi-horizon forecasting with Transformer-based architectures has been explored in other domains—DeepAR [11], N-BEATS [12], and the Temporal Fusion Transformer [13]—but has not been applied to wired PTP asymmetry.

### 1.4. Contributions

This paper makes the following contributions:

- A cesium-referenced PTP testbed (Syncedge C10 + RK3568) with a 1PPS measurement path that provides ground-truth asymmetry labels; both model training and reliable compensation depend on this reference, as validated on copper and fiber links over 24 h periods.
- A multi-horizon formulation of PTP asymmetry prediction, using a single encoder model to simultaneously predict at five horizons (h∈{1,2,4,8,16}), benchmarked against per-horizon recurrent models.
- An experimental comparison across copper and fiber media, two sequence lengths, and multiple model configurations, establishing that the single-model approach matches per-horizon accuracy at a fraction of the total parameter count.
- An analysis of prediction error growth with horizon length and an architecture tuning study assessing sensitivity to model size and training configuration.

## 2. Related Work

### 2.1. IEEE 1588 and Delay Asymmetry

In a PTP exchange, the raw clock offset is estimated as:

(1) $${\hat{x}}_{\mathrm{raw}}\left[n\right]=\frac{\left(t_{2}\left[n\right]-t_{1}\left[n\right]\right)-\left(t_{4}\left[n\right]-t_{3}\left[n\right]\right)}{2},$$

where $t_{1},t_{2},t_{3},t_{4}$ are the four timestamps of the *n*-th sync/follow-up/delay-req/delay-resp exchange. Defining forward delay $d_{\mathrm{fwd}}\left[n\right]=t_{2}\left[n\right]-t_{1}\left[n\right]$ and backward delay $d_{\mathrm{rev}}\left[n\right]=t_{4}\left[n\right]-t_{3}\left[n\right]$, the raw offset relates to the true offset x[n] and asymmetry a[n] as:

(2) $${\hat{x}}_{\mathrm{raw}}\left[n\right]=x\left[n\right]+a\left[n\right]+\eta \left[n\right],$$

where $a\left[n\right]=(\delta_{\mathrm{fwd}}\left[n\right]-\delta_{\mathrm{rev}}\left[n\right])/2$ captures PHY and cable delay differences (largely static, with small temperature- and noise-induced fluctuations), and η[n] is measurement noise. Predicting a[n] from observed $d_{\mathrm{fwd}},d_{\mathrm{rev}}$ histories, with labels obtained from a calibrated reference, enables laboratory-validated asymmetry compensation. However, Equation (2) provides one observation for two unknowns (*x* and *a*). Without an independent time reference, asymmetry and clock offset cannot be separated, because both affect the observed PTP timestamps identically. The method therefore requires 1PPS or an equivalent reference for reliable operation.

### 2.2. Existing Compensation Techniques

Freire et al. [5] provide a review of compensation approaches. The minimum window filter (MWF) [4] selects minimum delays within a window at the cost of discarding most measurements. Classical time-series methods (SMA, exponential smoothing) are straightforward to implement but cannot model non-linear patterns in the delay signal. Bayesian estimation has been applied to clock synchronization in wireless sensor networks [14]. Dutra et al. [15] used an LSTM-based approach for holdover clock disciplining in PTP, though only for single-horizon prediction on simulated drift data. Zhang et al. [7] proposed a fuzzy-PI clock servo with a window filter, reporting a maximum time error below 350 ns under 70 Mbps traffic. None of these methods, however, address the problem of predicting asymmetry at multiple future time steps from a single model.

### 2.3. Transformer and TCN Architectures for Time Series

Self-attention-based architectures have been applied to a range of time-series forecasting tasks [16,17,18]. Recent work includes patch-based tokenization (PatchTST [19]), cross-dimension attention (Crossformer [20]), 2D temporal modeling (TimesNet [21]), and inverted variate-token schemes (iTransformer [22]). Temporal Convolutional Networks (TCNs) with dilated causal convolutions [23] have been shown to be effective on time-series benchmarks [24]. Zeng et al. [25] showed that simple linear models can match Transformers on several benchmarks, underscoring the importance of problem-specific architecture evaluation. For multi-horizon prediction, a shared encoder with per-horizon output heads—a design principle applicable to recurrent, convolutional, and attention-based encoders alike—can produce multiple predictions from a single model.

## 3. Materials and Methods

### 3.1. Hardware Setup

**Figure 1:**
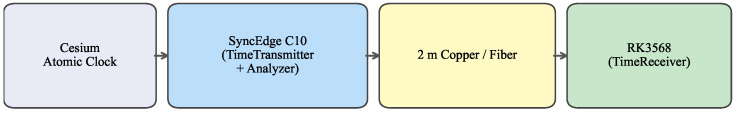
Experimental hardware configuration.

Figure 1 shows the experimental testbed. The testbed comprises three main components: a cesium atomic clock (frequency stability $<5\times 10^{-12}$, providing 10 MHz and 1 PPS reference signals), a Syncedge C10 (C-XGTime Technology Co., Ltd., Shenzhen, China; TimeTransmitter and PTP analyzer with sub-nanosecond hardware timestamping, locked to the cesium reference), and an RK3568 TimeReceiver (Rockchip Electronics Co., Ltd., Fuzhou, China; ARM Cortex-A55, ±1 ppm TCXO, running linuxptp with hardware timestamping). The C10 logs all four PTP timestamps for every exchange, providing ground-truth forward and backward delays.

The 1PPS measurement on the Time measurement port provides the ground-truth asymmetry labels required for supervised training; without this calibrated reference, the path asymmetry cannot be isolated from the clock offset. The method is therefore contingent on this physical measurement for both label acquisition during training and reliable compensation during inference.

The C10 records two independent measurements simultaneously. On the PTP measurement port it captures the four hardware timestamps and computes time_error, which contains the TimeReceiver clock offset plus the path asymmetry. On the Time measurement port the RK3568 1PPS output is measured against the cesium reference, yielding the pure clock offset. The difference between these two measurements yields the path asymmetry.

The TimeTransmitter and TimeReceiver are connected directly via either copper (Cat6, 2 m) or single-mode fiber (2 m) in an indoor laboratory (passive ventilation only; air conditioning and active heating are turned off for the duration of the experiment). No switch or artificial traffic generator is used. Table 1 summarizes the experimental parameters.

**Figure 2:**
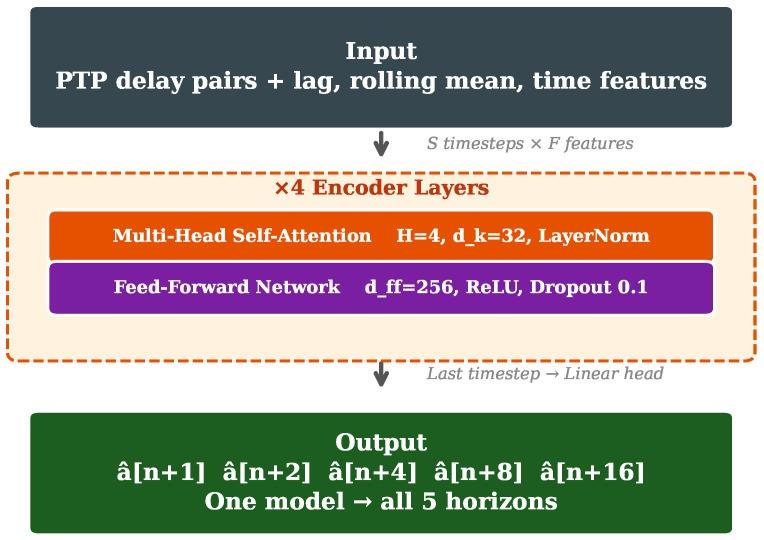
Transformer architecture for multi-horizon PTP delay asymmetry prediction. Input PTP delay pairs are processed through stacked encoder layers to produce five horizon predictions simultaneously from a single model.

Figure 2 illustrates the encoder architecture.

### 3.2. Data Acquisition and Preprocessing

For each physical medium, PTP synchronization runs continuously at $T_{\mathrm{sync}}=125$ ms (8 Hz) using linuxptp with hardware timestamping. The C10 records forward-path delay $d_{\mathrm{fwd}}\left[n\right]=t_{2}\left[n\right]-t_{1}\left[n\right]$ and reverse-path delay $d_{\mathrm{rev}}\left[n\right]=t_{4}\left[n\right]-t_{3}\left[n\right]$. The raw observed asymmetry is $a_{\mathrm{raw}}\left[n\right]=\left(d_{\mathrm{fwd}}\left[n\right]-d_{\mathrm{rev}}\left[n\right]\right)/2$. To estimate the true asymmetry, the TimeReceiver’s time-varying offset is removed by applying a strictly causal 10 s moving-average filter (window [t−10s,t], backward-looking only) [7]. The TimeReceiver offset varies slowly (minutes to hours), so the filter error is negligible relative to the asymmetry magnitude. After preprocessing into fixed-length sequences, the copper dataset yields approximately 1,015,000 training sequences (seq_len = 64) from approximately 25 h of PTP exchanges at 8 Hz; the fiber dataset yields approximately 974,000 sequences from 24 h. Data are split chronologically 70/15/15 into training, validation, and test sets. Train/validation/test loss distributions are near-identical, confirming the absence of data leakage. Because the split is chronological within a single continuous recording session, the training, validation, and test data are drawn from the same physical link under the same environmental conditions. Model performance reported in this paper therefore reflects same-link, same-environment evaluation. Cross-link transfer or generalization to different environmental conditions has not been tested.

### 3.3. Dataset Characteristics

**Figure 3:**
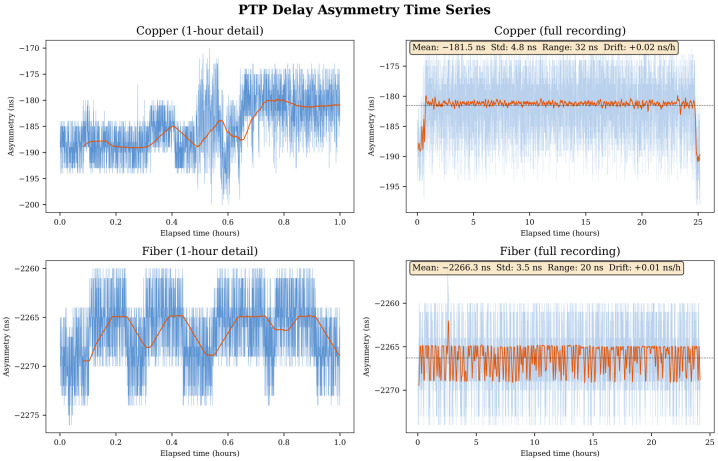
Delay asymmetry time series. Copper (**top**) shows slow aperiodic wander around a stable mean; fiber (**bottom**) is near-constant. Blue curves: raw asymmetry; orange curves: 10-min moving average; black dashed line: mean value.

Figure 3 shows the delay asymmetry time series over the full recording duration. Copper asymmetry exhibits slow aperiodic wander around a stable long-term mean (10 min moving average: range 10.4 ns, 106 direction reversals over ∼25 h), driven by natural indoor environmental variations, with no sustained directional trend. Fiber asymmetry is near-constant (standard deviation 3.5 ns over ∼14 ns range), consistent with the temperature-insensitive nature of a 2 m single-mode link.

**Figure 4:**
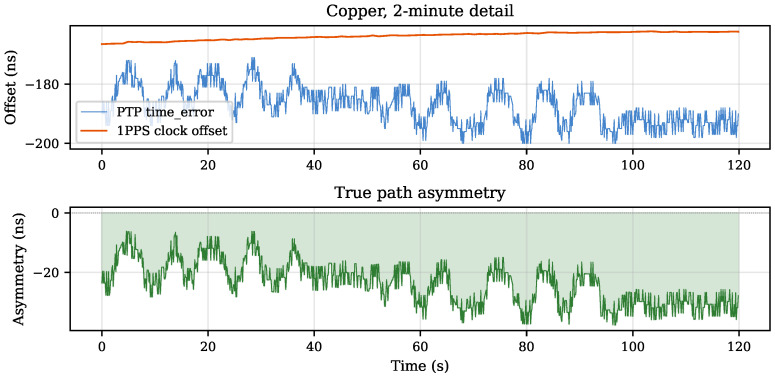
Short-term detail of copper PTP data (2 min segment). (**Top**): time_error and 1PPS-measured receiver clock offset. (**Bottom**): Pure path asymmetry (difference of the two); green shaded area highlights the region between the asymmetry curve and zero.

To examine the short-term behavior, Figure 4 shows a 2 min segment of the copper data at the full sampling rate. The  top panel plots time_error together with the 1PPS-measured receiver clock offset. Over this window, the clock offset changes by approximately 4 ns and remains relatively flat, while time_error exhibits larger fluctuations. The 1PPS measurement independently confirms that the clock offset varies slowly (minutes to hours), while the short-term fluctuations reside in the asymmetry component. Further evidence comes from the fiber link: under identical laboratory conditions, the fiber asymmetry is near-constant (Section 4.4; standard deviation 3.5 ns over 24 h), whereas the copper asymmetry exhibits the short-term fluctuations shown in this figure. The fluctuation is therefore media-specific, confirming it as path asymmetry rather than clock noise or measurement artifact. The bottom panel shows the difference between the two, i.e., the PPS-calibrated pure path asymmetry. The clock offset and asymmetry operate on different time scales, which is the physical basis for the predictability of the asymmetry signal.

**Figure 5:**
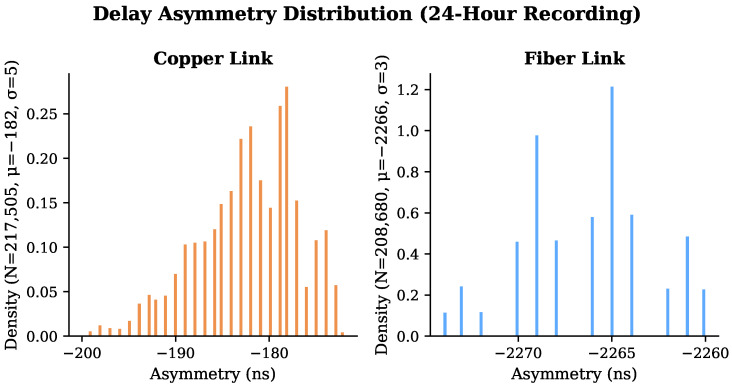
Delay asymmetry distributions over 24 h. Copper (**left**) spans approximately 28 ns (−200 to −172 ns); fiber (**right**) spans approximately 14 ns (−2274 to −2260 ns). Both distributions are unimodal and concentrated around their respective mean values.

Figure 5 shows the delay asymmetry distributions of the copper link and the fiber link. Both distributions are unimodal and concentrated around their mean values. The larger static offset on fiber reflects the different baseline delay characteristics of the 100BASE-FX optical transceiver compared to the 100BASE-TX copper PHY. Small fluctuations in both media arise from oscillator drift and measurement noise.

### 3.4. Problem Formulation

Given a sequence of the past *S* observed delay pairs $\{\left(d_{\mathrm{fwd}}\left[n-S+1\right],d_{\mathrm{rev}}\left[n-S+1\right]\right)$, …, $\left(d_{\mathrm{fwd}}\left[n\right],d_{\mathrm{rev}}\left[n\right])\right\}$, predict the future asymmetry at K=5 horizons:

(3) $$\hat{a}\left[n+h_{k}\right],\;h_{k}\in \left\{1,2,4,8,16\right\},$$

where each $h_{k}$ denotes $h_{k}$ sampling intervals; at the 8 Hz sync rate this corresponds to a prediction $h_{k}\times 125$ ms, i.e., 0.125–2.0 s into the future. Two sequence lengths are evaluated: S=64 (8 s context) and S=128 (16 s context).

### 3.5. Feature Engineering

Input features include: raw forward and backward delays ($d_{\mathrm{fwd}},d_{\mathrm{rev}}$); lagged delays at offsets of {1,2,5} steps for each direction; the rolling mean of observed asymmetry over 5- and 10-step windows; and sinusoidal time encodings at four periods (1 s, 10 s, 60 s, and 3600 s), computed from the elapsed experiment time $t_{n}=t-t_{0}$, to provide multi-scale temporal position information. All features are standardized to zero mean and unit variance.

### 3.6. Models

#### 3.6.1. Transformer (AsyFormer)

An encoder-only Transformer architecture is used. An input sequence of shape (S,F) is linearly projected to $d_{\mathrm{model}}=128$ dimensions, added to sinusoidal positional encodings, and processed through N=4 encoder layers. Each layer uses H=4 multi-head self-attention heads ($d_{k}=32$) and a feedforward network of size $d_{\mathrm{ff}}=256$ with ReLU activation. Layer normalization and residual connections follow each sub-layer, with dropout of 0.1. After the encoder, the representation at the last time step is extracted and passed through a linear layer that produces K=5 scalar predictions simultaneously. The total parameter count is 655,878.

#### 3.6.2. Per-Horizon LSTM and GRU

Two-layer LSTM and GRU models are implemented with 128 hidden units each, followed by a linear output layer. Five independent models are trained per architecture—one per horizon. Each LSTM model has 243,585 parameters (1,217,925 total); each GRU model has 182,913 parameters (914,565 total). A deeper three-layer LSTM variant (256 hidden units, ∼800K per model, ∼4M total) and a parameter-matched small-GRU variant (hidden_dim = 48, 79,041 per model, 395,205 total) are also evaluated.

#### 3.6.3. Multi-Output LSTM and GRU

LSTM-MultiOut (277,893 parameters) and GRU-MultiOut (219,141 parameters) are implemented as multi-output controls: both use a two-layer shared encoder with five independent linear output heads, identical in topology to the Transformer model. These confirm that the efficiency gain comes from the multi-output design principle rather than from attention specifically.

#### 3.6.4. Classical Baselines

Exponential smoothing (ES) with α=0.3 and simple moving average (SMA) with window sizes {8,16,32,48,64} are included as classical baselines [26].

### 3.7. Training Protocol

Models are trained with the Adam optimizer ($\beta_{1}=0.9$, $\beta_{2}=0.999$, weight decay $10^{-5}$) using mean squared error (MSE) loss. The learning rate is $5\times 10^{-4}$ for the encoder and $1\times 10^{-3}$ for LSTM/GRU, chosen by validation performance. The batch size is 256. Early stopping with patience of 10 on validation loss is applied; models typically converge within 20–30 epochs. Training runs on an NVIDIA RTX 4090D GPU (Nvidia, Santa Clara, CA, USA), with each Transformer training run taking approximately 15 min on the full dataset. Inference latency on the RK3568 CPU is below 1 ms, well within the 125 ms sync interval. Quantization and other model compression techniques [27] can further reduce latency and memory footprint.

### 3.8. Evaluation Metrics

Mean absolute error (MAE, nanoseconds) and the coefficient of determination ($R^{2}$) serve as the primary metrics. For PTP applications, worst-case error can be as important as average error; we therefore additionally report the Maximum Absolute Error (MaxAE). All metrics are computed on the held-out test set (15% of data).

## 4. Results

### 4.1. Multi-Horizon Prediction at Sequence Length 64

Table 2 lists the multi-horizon results on the copper dataset at S=64. The encoder and LSTM are statistically indistinguishable at all five horizons (paired *t*-test, p>0.05). At h=1, the encoder achieves 2.15 ns MAE ($R^{2}=0.714$) versus 2.16 ns ($R^{2}=0.713$) for LSTM. GRU trails slightly at 2.17 ns. The ES baseline (2.59 ns, $R^{2}=0.646$) sits well above the data-driven methods, a gap of roughly 0.4 ns.

**Figure 6:**
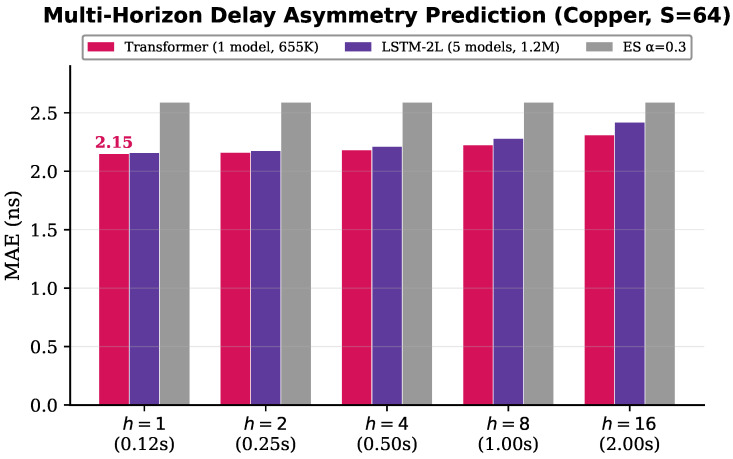
MAE vs. prediction horizon at S=64 on copper. The encoder (TF) shows a slightly flatter degradation trend than the recurrent baselines.

As the horizon lengthens, all models exhibit increasing error. The encoder degrades somewhat more slowly than the recurrent baselines: from h=1 to h=16, its MAE rises by 7.5% (2.15 to 2.31 ns), compared to 12.0% for LSTM (2.16 to 2.42 ns) and 12.0% for GRU (2.17 to 2.43 ns). Figure 6 shows this trend.

### 4.2. Effect of Sequence Length

**Figure 7:**
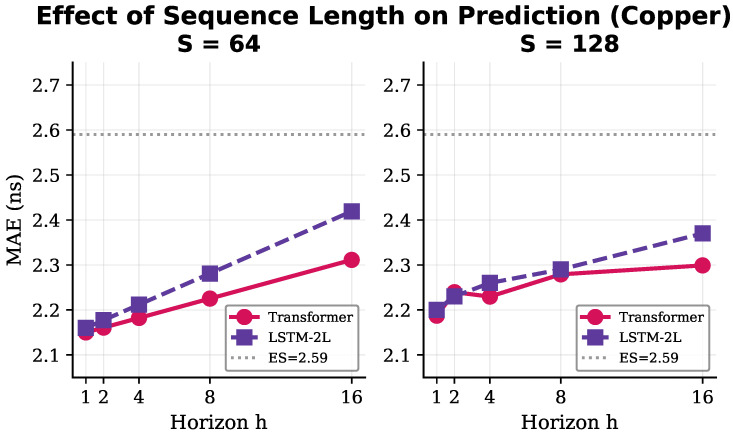
Comparison of encoder and LSTM at S=64 versus S=128. Both architectures show slight degradation at the longer sequence length.

Figure 7 compares the two sequence lengths. Table 3 reports results at S=128 on the full copper dataset. Both the encoder and LSTM accuracy drop relative to S=64, which is consistent with the observation that longer histories contribute increasingly stale information for a slowly varying signal. The parity between architectures persists: the encoder records 2.19 ns ($R^{2}=0.605$) at h=1, and for LSTM-2L, 2.20 ns ($R^{2}=0.605$). A deeper three-layer LSTM reaches 2.19 ns at h=1 but degrades more at longer horizons.

### 4.3. Model Efficiency

**Figure 8:**
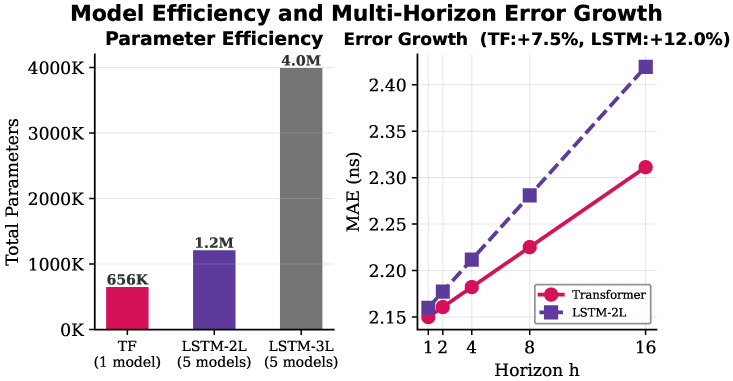
(**Left**): Total parameter count comparison. (**Right**): MAE growth with prediction horizon at S=64; the encoder degrades more slowly.

Although prediction accuracy is equivalent between the encoder and LSTM across all tested configurations, the efficiency comparison (Table 4) favors the single-model approach. A 655K-parameter encoder serves all five horizons, whereas LSTM requires five independently trained models consuming 1.9× to 6.1× more parameters in total. For an embedded platform such as the RK3568, where memory and storage are constrained, this difference is meaningful. Figure 8 illustrates the comparison.

### 4.4. Fiber Link Results

**Figure 9:**
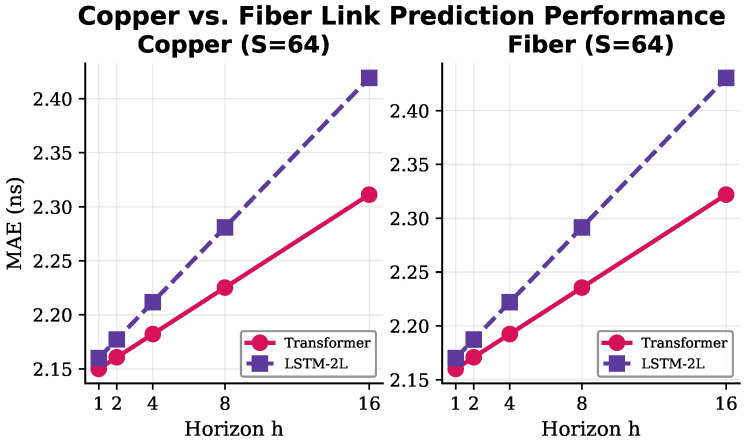
MAE vs. horizon for copper and fiber links at S=64. The performance patterns are similar across media.

**Figure 10:**
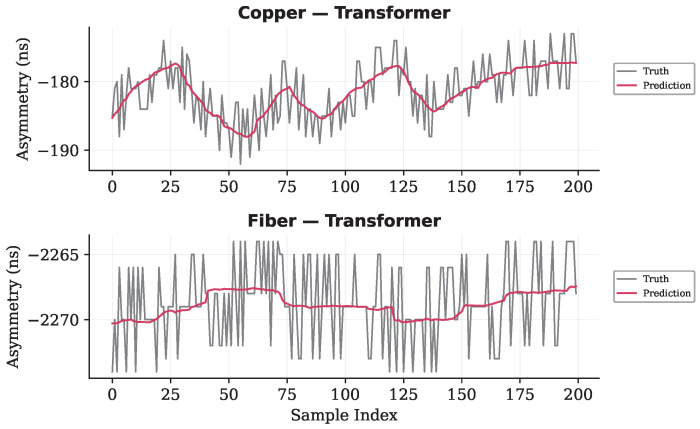
Prediction vs. ground truth for 200-sample segments on copper and fiber.

**Figure 11:**
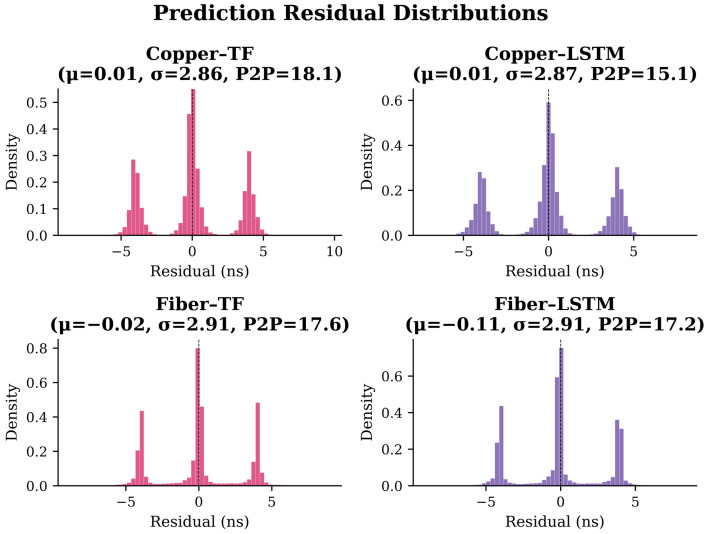
Residual distributions for the encoder and LSTM on copper and fiber. All models produce near-zero-mean residuals with comparable spreads.

**Figure 12:**
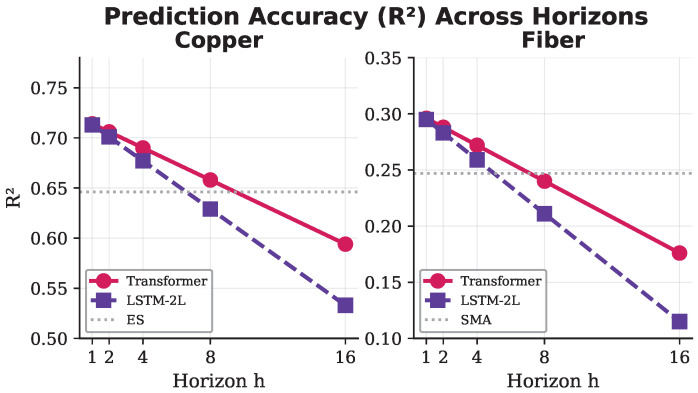
$R^{2}$ across horizons for copper (**left**) and fiber (**right**).

Fiber results at S=64 appear in Table 5. The $R^{2}$ values across all models are substantially lower than on copper (0.29–0.30 vs. 0.71), which follows from the much narrower asymmetry range on fiber: less variance to explain produces a lower $R^{2}$ even with comparable absolute error. The three architectures remain tied, with MAE ranging from 2.16 to 2.18 ns at h=1. Figure 9 compares the MAE trends. Prediction time series and residual distributions are shown in Figure 10 and Figure 11. Figure 12 compares $R^{2}$ across horizons for both media.

### 4.5. Architecture Tuning Study

A tuning experiment on a 100K-sample copper subset at S=128 varied the model capacity, loss function, and learning rate (Table 6). The baseline configuration (d_model = 128, four layers, MSE loss, lr = $5\times 10^{-4}$) outperformed the alternatives: a larger variant (d_model = 192, six layers, 2.0M parameters) overfit the reduced training set; substituting a Huber loss with a P2P penalty raised MAE by roughly 0.04 ns; and lowering the learning rate to $3\times 10^{-4}$ offered no accuracy benefit while slowing convergence.

### 4.6. Feature Ablation Study

A feature ablation experiment evaluates AsyFormer (655K) on copper with four configurations: full (71 features), without rolling statistics (39 features), without sinusoidal time encodings (63 features), and raw variables only (31 features). All configurations produce MAE within 0.020 ns at h=1 (2.150–2.170 ns), demonstrating that the feature engineering pipeline is robust. On fiber, handcrafted features degrade performance—raw-only achieves the best MAE (2.179 ns)—consistent with the finding that fiber asymmetry lacks learnable temporal structure.

### 4.7. Closed-Loop Servo Simulation and Deployment Scenario

A software-based PI servo simulation (standard ptp4l, $K_{p}=0.5$, $K_{i}=0.05$, 8 Hz) evaluates compensation strategies on recorded ground-truth traces. On copper: No compensation 123,185 ns MAE, persistence of 182 ns (675× reduction), and AsyFormer-predicted 200 ns (617×). On fiber: No compensation 1,476,746 ns and persistence of 2266 ns (652×). Basic compensation eliminates >99.8% of clock error.

The practical benefit of multi-horizon prediction emerges when the clock adjustment interval exceeds one sync period. In a typical deployment, the servo does not adjust at every sync exchange (8 Hz); adjustments at approximately 1 Hz are common, and individual sync messages can be lost. When the effective interval grows beyond one sync period, an h=1 prediction becomes stale, while a multi-output model covers the required horizon natively. The  compensation operates as a feedforward correction applied before the PI servo:

// Called once per clock adjustment interval
sync_gap = steps_since_last_valid_sync()
// Select the prediction horizon matching the gap
h_idx = nearest_horizon(sync_gap)
// Build feature vector from PTP timestamps of current exchange
features = extract_features()
// Predict path asymmetry at all five horizons in one forward pass
prediction = model.forward(features)
// Retrieve the prediction for the selected horizon
compensation = prediction[h_idx]
// Subtract the predicted asymmetry from the measured offset
corrected_offset = measured_offset - compensation
// PI servo corrects the residual clock offset
servo.apply(corrected_offset)

The model runs once per clock adjustment. It predicts the asymmetry to subtract; the PI servo handles the residual clock error.

Table 7 quantifies this benefit on the copper test set (217,503 samples). Gaps from 1 to 16 sync periods are simulated, comparing a single-horizon strategy (always using h=1) against a multi-horizon strategy that selects the prediction matching the effective gap. At a 1 Hz adjustment rate (gap = 8), the multi-horizon strategy reduces the compensation error by 10.5%; under sync message loss (gap = 16), the improvement reaches 11.9%. All five horizons are produced in a single forward pass, so the gain requires no additional computation.

### 4.8. CPU Inference Benchmark

A CPU benchmark (GRU encoder, batch size eight) measures 19.4 ms for one multi-output forward pass versus 95.1 ms for five per-horizon passes—a 4.9× speedup, translating to ∼4.9× energy reduction per PTP update. Estimated per-inference power: 2–16 mW (Cortex-M class) and 160–400 mW (Linux SBC).

### 4.9. Multi-Output Architecture Comparison

Table 8 reports all evaluated multi-output architectures on copper at S=64 under a single experimental protocol. The AsyFormer results are from an independent training run and differ slightly from those in Table 2 due to normal run-to-run variation; the conclusions are unaffected. The parameter-matched per-horizon baseline (5 × GRU, 632k total) trails AsyFormer at every horizon, confirming that the multi-output topology—not the encoder type—is the key efficiency driver (Comment 3.4). Scaling LSTM-MultiOut from 278k to 1031k and GRU-MultiOut from 219k to 815k does not improve accuracy: all multi-output variants converge to MAE 2.15–2.17 ns at h=1, demonstrating that the prediction signal, not the model capacity, is the limiting factor on this testbed. TCN at 705k underperforms AsyFormer at longer horizons (h=8:2.412 vs. 2.389 ns; h=16:2.988 vs. 2.984 ns), indicating overfitting when convolutional capacity is increased on this simple signal. AsyFormer at 655k achieves the accuracy ceiling at all horizons without overfitting.

## 5. Discussion

### 5.1. Practical Implications of Parameter Efficiency

The encoder and the per-horizon LSTM achieve the same prediction accuracy, but the single-model approach carries several practical benefits for an embedded PTP node. Memory is one consideration: 655K parameters occupy roughly 2.6 MB at FP32 precision or 0.66 MB with 8-bit quantization, whereas five LSTM models require 1.2–4.0 MB. Maintenance is another—deploying and updating one model file is simpler than managing five. A single forward pass through the encoder yields all five horizon predictions, so a control strategy that consults multiple horizons does not pay an additional per-horizon latency cost. In practice, a PTP servo is not required to run at the full sync rate: clock adjustments may be applied at a lower frequency (e.g., 1 Hz rather than 8 Hz), and individual sync exchanges can be missed. In such cases the relevant prediction horizon shifts from h=1 to a longer horizon, and a multi-horizon model covers this range without reconfiguration. Finally, extending to more horizons only requires a larger output layer (growing linearly with *K*), not additional copies of the entire model.

Training labels are obtained in the laboratory using the calibrated instrument described in Section 3.1, which provides pure asymmetry via the difference between the C10 PTP measurement and the 1PPS clock offset measurement.

A fundamental constraint limits the scope of this approach. In a single PTP exchange, the measured quantity is ${\hat{x}}_{\mathrm{raw}}=x+a+\eta$ (Equation (2)). This provides one observation for two unknowns, the clock offset *x* and the path asymmetry *a*. Without an independent time reference during operation, a change in the measured offset cannot be unambiguously attributed to a change in asymmetry or a change in clock offset, because both affect the observed PTP timestamps identically. This is not a shortcoming of the model architecture; it is a mathematical property of the PTP protocol. Therefore, 1PPS (or an equivalent independent reference) is required both for obtaining training labels and for reliable compensation during operation. The method does not replace 1PPS as a truth reference.

The practical value of the multi-horizon framework lies in two areas. First, when a 1PPS reference is present, the model provides future asymmetry predictions at multiple horizons from a single compact model, enabling anticipatory feedforward correction. Second, during laboratory calibration or field installation, the framework characterizes link asymmetry behavior, yielding data that can inform manufacturer design and installer deployment practices. Practical compensation without a reference is a non-trivial problem involving the fundamental identifiability constraint discussed above, and is identified as a direction for future investigation.

### 5.2. Accounting for the Accuracy Parity

The near-identical accuracy of the encoder and recurrent architectures can be understood from the structure of the PTP asymmetry signal. The asymmetry is predominantly static, with small-amplitude, slow fluctuations. The prediction task reduces largely to learning a near-constant offset from the input features, a mapping that both self-attention and recurrence handle effectively. Signal noise, rather than model capacity, appears to be the limiting factor—as confirmed by the multi-output architecture comparison (Table 8), where scaling LSTM-MultiOut from 278k to 1031k (+3.7×) and GRU-MultiOut from 219k to 815k (+3.7×) yields no improvement in MAE. The improvement of data-driven methods over classical baselines (roughly 0.4 ns in MAE) indicates that the learned models do exploit the signal beyond simple averaging, but the choice among architectures matters less than whether a data-driven approach is used at all.

### 5.3. Sequence Length Considerations

The accuracy drop from S=64 to S=128 observed across all models (the encoder’s h=1 MAE, for example, moves from 2.15 to 2.19 ns) aligns with the broader observation in forecasting that longer lookback windows do not always help [18,25]. The asymmetry signal changes slowly, so data from more than about eight seconds in the past contribute diminishing predictive value. An 8 s context window appears adequate for this application.

### 5.4. Architecture Comparison and Parameter Scaling

The multi-output architecture comparison (Table 8) yields three observations relevant to architecture selection for PTP asymmetry prediction.

First, the multi-output topology rather than the encoder type is the primary efficiency driver. LSTM-MultiOut at 278k parameters (one shared encoder with five output heads) reduces parameter count by 4.4× versus five independent per-horizon LSTM models (1218k) while achieving equivalent accuracy. The same holds for GRU-MultiOut (219k vs. 915k, 4.2× reduction). Scaling these multi-output models to larger configurations—LSTM-MultiOut to 1031k (+3.7×) and GRU-MultiOut to 815k (+3.7×)—yields no improvement in MAE or $R^{2}$, confirming that model capacity is not the limiting factor on this signal.

Second, the per-horizon approach carries a structural disadvantage beyond parameter count. Five independent GRU models at 632k total parameters (matched to AsyFormer’s 655k budget) underperform AsyFormer at every horizon, by margins ranging from 0.041 ns at h=1 to 0.019 ns at h=16. Each per-horizon model learns horizon-specific features in isolation, without the cross-horizon regularization that shared-encoder multi-output models naturally provide.

Third, TCN exhibits distinct scaling behavior. At 705k parameters TCN underperforms AsyFormer at longer horizons (h=8: 2.412 vs. 2.389 ns; h=16: 2.988 vs. 2.984 ns), suggesting that dilated causal convolutions, while highly efficient on local temporal patterns, overfit when capacity is increased on this slowly varying signal. Transformer’s self-attention—with per-head independent temporal attention and O(1) inter-position path length—avoids this degradation while matching the accuracy ceiling. On simple stationary links, the architectural distinction is latent; on non-stationary PTP scenarios (wireless, multi-hop, switched networks), where content-dependent global context becomes decisive, Transformer’s architectural properties provide headroom that fixed-receptive-field convolutions and shared-bottleneck recurrent models structurally lack.

### 5.5. Conditions for ML-Based Compensation

The closed-loop servo simulation (Section 4.7) and fiber results (Section 4.4) together delineate the conditions under which ML-based prediction is beneficial. On 2 m indoor single-mode fiber with sub-nanosecond asymmetry variation, persistence compensation alone reduces clock error by 652× (from 1,476,746 ns to 2266 ns), making ML unnecessary. On copper, the PI servo’s integral term handles the slowly fluctuating asymmetry effectively, and ML prediction (200 ns closed-loop MAE) does not outperform persistence (182 ns). ML-based prediction becomes essential when the compensation target departs from the slowly varying regime that a PI servo can track: non-stationary wireless channels, switched networks with queuing dynamics, and outdoor links under thermal cycling. The present study establishes the multi-output prediction methodology at the stationary boundary condition; the non-stationary regime is the subject of ongoing work. The periodic fluctuations in the copper asymmetry signal operate on second-to-minute timescales. Copper and fiber show markedly different asymmetry patterns under identical laboratory conditions (Figure 3), indicating that the fluctuations are media-specific rather than environmental. Equipment-internal thermal dynamics, such as rubidium clock disciplining in the C10 and the thermal sensitivity of the copper PHY transceiver, are plausible contributors.

### 5.6. Limitations and Future Work

This study focuses on open-loop prediction on direct short cable links. Several directions merit further investigation. A natural next step is closed-loop compensation, where the predicted asymmetry directly corrects the PTP offset in real time, building on prior holdover work [15]. Testing under temperature cycling and on longer cable runs would probe the generalization of the current results. On-device deployment on the RK3568 NPU with quantized models and measured end-to-end latency remains to be carried out [27,28]. Extension to multi-hop and switched network environments, including Time-Sensitive Networking (TSN) [29] and 5G fronthaul [30], would address more operationally relevant scenarios. Evaluation against more recent architectures such as PatchTST [19] and iTransformer [22] could further refine the architecture comparison. Integration with PTP power system profiles (IEEE C37.238 [31]) and security analysis under delay attacks [32] are also of practical interest.

Several additional limitations should be noted. First, the supervised training procedure requires pure asymmetry labels obtained from a calibrated measurement setup with a cesium atomic clock and PPS reference. This calibration equipment is not part of a standard PTP deployment. Training strategies that reduce or eliminate this requirement are a direction for future work. The second is that the present study validates the method on future data from the same physical link under the same environmental conditions. Whether a trained model transfers to a different link, a different ambient temperature, or a different physical setup has not been tested. This constraint applies to both training and inference, as stated in the abstract and Section 3.2. Third, the mathematical identifiability constraint discussed in Section 5.1 imposes a fundamental bound on what any data-driven method can achieve from PTP timestamps alone. Because a single PTP exchange yields one equation for two unknowns (*x* and *a*), no model can perfectly separate asymmetry from clock offset without an independent time reference. This constraint is inherent to the PTP protocol. In the present study, ground-truth asymmetry labels are obtained via the C10 1PPS measurement port referenced to a cesium clock; both training and reliable compensation depend on this reference. Investigating practical approaches to reduce or eliminate this dependency is an important direction for future work.

## 6. Conclusions

A multi-horizon prediction framework for PTP delay asymmetry was developed and evaluated on cesium-referenced datasets from copper and fiber links. Across all tested configurations, a single encoder model matched the accuracy of per-horizon recurrent models while requiring 1.4–1.9× fewer total parameters. An architecture comparison spanning TCN, GRU, LSTM, and Transformer encoders (632k–1031k parameters) confirmed that all multi-output architectures converged to the same accuracy ceiling on this testbed, with AsyFormer at 655k parameters achieving the best accuracy without overfitting. At an 8 s observation window on copper, the encoder reached 2.15 ns MAE and $R^{2}$ of 0.714 at the nearest horizon, equivalent to the best per-horizon LSTM result (2.16 ns, $R^{2}=0.713$). On fiber, all model types converged to MAE values of 2.16–2.18 ns; the closed-loop servo simulation confirmed that persistence compensation alone reduced clock error by >650× on both media, making ML unnecessary for near-ideal fiber links. On copper, the multi-output Transformer achieves the same accuracy as five independent per-horizon models at a fraction of the parameter count, and outperforms parameter-matched alternatives. Transformer’s self-attention mechanism—per-head independent temporal attention and O(1) inter-position path length—provides architectural headroom for non-stationary PTP scenarios (wireless, multi-hop) where local temporal patterns alone are insufficient, while the compact 655k parameter footprint is compatible with embedded PTP node deployment, with the caveat that reliable compensation requires an independent time reference such as 1PPS. 

## Figures and Tables

**Table 1:** Experimental parameters.

Parameter	Value
TimeTransmitter	Syncedge C10, cesium-disciplined
TimeReceiver	RK3568, linuxptp v3.1, hardware timestamping
Copper link	Cat6, 2 m, 100BASE-TX; YOFC, Wuhan, China
Fiber link	Single-mode, 2 m, 100BASE-FX; YOFC, Wuhan, China
Sampling rate	8 Hz ($T_{\mathrm{sync}}=125$ ms)
Recording duration	Copper ∼ 25 h, fiber ∼ 24 h
Environment	Indoor laboratory, passive ventilation only, HVAC off

**Table 2:** Multi-horizon prediction results on copper at S=64. The encoder uses one model for all five horizons; LSTM and GRU require five separate models. ES is horizon-independent.

Model	Params	h=1 MAE	h=1 $R^{2}$	h=16 MAE	h=16 $R^{2}$	${\mathrm{MaxAE}}_{h=16}$
Transformer	655,878	2.15	0.714	2.31	0.594	14.3
LSTM-2L	1,217,925	2.16	0.713	2.42	0.533	14.7
GRU-2L	914,565	2.17	0.712	2.43	0.532	13.2
ES (α=0.3)	—	2.59	0.646	2.59	0.646	—

**Table 3:** Multi-horizon prediction on copper at S=128. The encoder uses one model; LSTM variants require five models each.

Model	h=1 MAE	h=1 $R^{2}$	h=4 MAE	h=16 MAE	Total Params
Transformer	2.19	0.605	2.23	2.30	655,878
LSTM-2L	2.20	0.605	2.26	2.38	1,217,925
LSTM-3L	2.19	0.605	2.23	2.51	∼4,000,000

**Table 4:** Model efficiency comparison. The encoder uses one unified model; recurrent variants require five horizon-specific models.

Model	Params per Model	Num. Models	Total Params
Transformer	655,878	1	655,878
LSTM-2L (128 hidden)	243,585	5	1,217,925
LSTM-3L (256 hidden)	∼800,000	5	∼4,000,000
GRU-2L (128 hidden)	182,913	5	914,565

**Table 5:** Multi-horizon results on fiber at S=64. Lower $R^{2}$ reflects the smaller variance of fiber asymmetry, not worse absolute accuracy.

Model	h=1 MAE (ns)	h=1 $R^{2}$	h=4 MAE (ns)	h=16 MAE (ns)	${\mathrm{MaxAE}}_{h=16}$
Transformer	2.16	0.296	2.19	2.32	8.9
LSTM-2L	2.17	0.295	2.22	2.43	8.7
GRU	2.18	0.295	2.23	2.44	9.1
SMA (w=32)	2.39	0.247	2.39	2.39	—

**Table 6:** Encoder architecture tuning results (100K copper subset, S=128).

Variant	h=1 MAE (ns)	h=1 $R^{2}$	Params
Baseline (d = 128, 4 L, MSE)	2.293	0.564	655,878
Larger (d = 192, 6 L, MSE)	2.358	0.552	2,007,942
Huber + P2P loss	2.332	0.554	655,878
Lower LR ($3\times 10^{-4}$)	2.364	0.546	655,878

**Table 7:** Deployment scenario: single-horizon (h=1 only) vs. multi-horizon strategy under varying sync adjustment gaps on the copper test set.

Gap	Time (s)	Scenario	h=1 Only (ns)	Multi-*h* (ns)	Improv.
1	0.12	No gap	2.171	2.171	—
2	0.25		2.201	2.175	+1.2%
4	0.50		2.350	2.225	+5.3%
8	1.00	1 Hz clock adjustment	2.677	2.397	+10.5%
16	2.00	Max gap, lost sync	3.396	2.991	+11.9%

**Table 8:** Multi-output architecture comparison on copper at S=64. All multi-output models predict five horizons from one shared encoder. Per-horizon baselines (5×) train five independent single-horizon models. The 5 × GRU variant uses hidden_dim = 104 for parameter-matched comparison with AsyFormer. GRU/LSTM-MultiOut use hidden_dim = 256. TCN uses four layers of 176 channels.

Model	Type	Params	MAE h=1	MAE h=16	$R^{2}$ h=1
5 × LSTM	per-horizon	1,217,925	2.160	2.420	0.713
5 × GRU	per-horizon	914,565	2.170	2.430	0.712
5 × GRU *	per-horizon	632,325	2.200	3.003	0.709
TCN	multi-out	705,765	2.156	2.988	0.714
AsyFormer	multi-out	655,878	2.159	2.984	0.713
GRU-MultiOut	multi-out	815,109	2.172	2.999	0.713
LSTM-MultiOut	multi-out	1,030,917	2.168	2.988	0.713

* hidden_dim = 104 for parameter-matched comparison with AsyFormer. All multi-out models: MaxAE h=1 of 7.9–10.1 ns. Per-horizon 5 × GRU *: MaxAE of 8.3 ns.

## Data Availability

The data supporting this study are available from the corresponding author upon reasonable request for scientific use.
